# Use of urgent, emergency and acute care by mental health service users: A record-level cohort study

**DOI:** 10.1371/journal.pone.0281667

**Published:** 2023-02-13

**Authors:** Jen Lewis, Scott Weich, Colin O’Keeffe, Tony Stone, Joe Hulin, Nicholas Bell, Mike Doyle, Mike Lucock, Suzanne Mason

**Affiliations:** 1 School of Health and Related Research, University of Sheffield, Sheffield, United Kingdom; 2 Sheffield Health and Social Care NHS Foundation Trust, Sheffield, United Kingdom; 3 South West Yorkshire Partnership NHS Foundation Trust, Wakefield, United Kingdom; 4 University of Huddersfield, Huddersfield, United Kingdom; Istanbul Bakirkoy Prof Dr Mazhar Osman Ruh Sagligi ve Sinir Hastaliklari Egitim ve Arastirma Hastanesi, TURKEY

## Abstract

**Background:**

People with serious mental illness experience worse physical health and greater mortality than the general population. Crude rates of A&E attendance and acute hospital admission are higher in people with serious mental illness than other hospital users. We aimed to further these findings by undertaking a standardised comparison of urgent and emergency care pathway use among users of mental health services and the general population.

**Methods:**

Retrospective cohort analysis using routine data from 2013–2016 from the CUREd dataset for urgent and emergency care contacts (NHS 111, ambulance, A&E and acute admissions) and linked mental health trust data for Sheffield, England. We compared annual age- and sex-standardised usage rates for each urgent and emergency care service between users of mental health services and those without a recent history of mental health service use.

**Results:**

We found marked differences in usage rates for all four urgent and emergency care services between the general population and users of mental health services. Usage rates and the proportion of users were 5–6 times and 3–4 times higher in users of mental health services, respectively, for all urgent and emergency care services. Users of mental health services were often more likely to experience the highest or lowest acuity usage characteristics.

**Conclusions:**

Current users of mental health services were heavily over-represented among urgent and emergency care users, and they made more contacts per-person. Higher service use among users of mental health services could be addressed by improved community care, more integrated physical and mental health support, and more proactive primary care. A complex pattern of service use among users of mental health services suggests this will need careful targeting to reduce avoidable contacts and optimise patient outcomes.

## Introduction

People with long-term physical health conditions (LTCs) experience rates of mental health problems at least twice that of the general population [1,2], and up to one-half of those with a mental health condition also have at least one LTC [3,4]. People with serious mental illness (SMI) (including schizophrenia or bipolar disorder) are five times more likely to have 3 or more LTCs [2,3] and die on average 10–20 years earlier than those without SMI [5,6], most often from cardiovascular and respiratory causes. People with SMI are less active, smoke more and have less healthy diets than those without SMI [2,7–9]. These risks are exacerbated by antipsychotic medication [10].

Although people with SMI access primary care more often than the general population [11], they are less likely to receive proactive and preventive interventions [12,13] and more likely to use acute and urgent medical care [4,14,15]. A study by the Nuffield Trust examined Hospital Episode Statistics (HES) data for England between 2009 and 2014 [14]. Three cohorts were identified: (i) a mental health cohort (n = 535,739) who used inpatient or outpatient mental health care; (ii) a SMI subgroup (n = 50,987) with at least one inpatient mental health episode and a diagnosis of schizophrenia, bipolar disorder or psychosis; and (iii) a physical health (PH) cohort (n = 13,141,421) who used hospital services but had no record of mental health service use. Rates for A&E attendances were over 3 times higher than the PH cohort among the mental health and SMI cohorts, while rates of emergency admissions were 4.9 and 6.7 times higher than the PH cohort in the mental health and SMI cohorts, respectively.

Improving outcomes and reducing avoidable admissions among people who have experienced mental illness are major policy priorities and sources of potential savings [16,17]. However, despite evidence about A&E presentations and acute admissions, we know little about use of care in different parts of the urgent and emergency care (UEC) pathway by people with mental health problems. This evidence will highlight areas where there is increased use of urgent, emergency and acute care services, to indicate targets for further investigation to develop interventions to improve outcomes.

The current study was a collaboration between the Urgent and Emergency Care and Mental Health and Multi-morbidity themes of the NIHR Applied Research Collaborative Yorkshire and Humber (ARC YH) programme.

### Aims

Our primary aim was to estimate rates of urgent, emergency and acute care use among people with a recent history of using secondary care mental health services (a proxy for SMI) and to compare these with rates among those who have not used mental health services in the past year in a northern city in England, standardised for age and sex. We also sought to compare additional informative indicators at different stages of the acute and urgent care pathway, such as length of acute hospital admissions and rates of non-urgent A&E attendances.

## Methods

### Study design

Retrospective cohort study.

### Setting

Sheffield, South Yorkshire. Sheffield is the seventh largest city in the UK, with an estimated population of approximately 597,000 [18].

### Participants

We identified two groups of individuals: UEC service users within the Sheffield Clinical Commissioning Group (CCG) who are current or recent users of mental health services (UMHS) and those who have not used mental health services for at least the previous 12 months. Participants were restricted to individuals between the ages of 15 and 105. The lower age limit was chosen to align with WHO standard age categories [19] and the upper limit to avoid errors associated with documentation.

### Data sources

#### Use of urgent and emergency care (UEC)

We used routinely collected patient data held in the University of Sheffield Centre for Urgent and Emergency Care research database (CUREd) [20–22]. This database holds linked record-level data from the Yorkshire and the Humber region on NHS 111 calls (telephone helpline), emergency ambulance incidents, A&E attendances, emergency admissions to hospital. Mental health service data from Sheffield Health and Social Care NHS Foundation Trust are also held. Data were available from 2011–2017. A common patient identifier enabled data linkage across datasets. Further details regarding the cleaning, linkage and associated methodological issues are available in a previous publication [22]. JL and TS had access to the data.

The NHS 111 calls dataset comprises call data for all calls to the NHS 111 telephone service operated by Yorkshire Ambulance Service NHS Trust (YAS) covering the Yorkshire and Humber region. Data included items relating to caller demographics and the triage outcomes of calls as recorded at the time of the call. Data were available from April 2013 to March 2017 and includes 4,789,273 distinct NHS 111 calls.

The ambulance incidents dataset records emergency calls (at incident level) to the emergency ambulance service operated by Yorkshire Ambulance Service NHS Trust covering the Yorkshire area. Data were available from April 2012 to March 2017 and include 4,382,835 distinct ambulance incidents.

The Accident & Emergency (A&E) attendances dataset comprises patient-level activity records at all Emergency Departments, Urgent Care Centres, and Minor Injury Units operated by acute NHS hospital trusts in Yorkshire and Humber. The data mainly comprise (direct or derived) items mandated by the national Commissioning Data Set (CDS). These data are generated by patient administration systems within each Trust. Data are available from April 2011 to March 2017, consistently from April 2013 and include 9,787,270 distinct attendances.

The Admitted Patient Care (APC) episodes dataset comprises patient records for care under each responsible healthcare professional at all acute hospital trusts in Yorkshire and Humber as the result of an emergency admission to that hospital, and specifically items mandated by the national Commissioning Data Set (CDS). These data are generated by patient administration systems within each Trust. Data are available from April 2011 to March 2017 and include 4,586,889 distinct episodes. The Provider Spell dataset is derived from the APC episodes dataset, where episodes (provider spells) are collapsed into continuous periods of care under a single (Hospital Trust) provider. Data are available from April 2011 to March 2017 and include 3,288,757 distinct provider spells.

All datasets included Index of Multiple Deprivation (IMD) decile. Postcodes were classified into IMD deciles using 2015 English Indices of Deprivation, which were obtained from the UK Government website [23].

### Mental health service use

The CUREd database includes data on all service users open to Sheffield Health and Social Care NHS Foundation Trust (SHSC) from 2011 to 2017. SHSC provides all specialist secondary mental health care for the population of Sheffield. These data include information on diagnoses, Health of the Nation Outcome Scales (HoNOS), mental health care clusters, community care episodes, inpatient spells and mental health detentions. Duplicate entries and records outside of the study period were removed, and participants with an address outside of the Sheffield CCG boundary were excluded. Participants were included irrespective of their use of urgent, emergency and acute care.

### Ethics statement

The CUREd database has approval from the Leeds East National Health Service (NHS) Research and Ethics Committee (18/YH/0234) and from the NHS Health Research Authority’s Confidentiality Advisory Group (18/CAG/0126). The need for direct patient consent was waived by CAG but patients were able to opt-out following a process detailed on posters in patient-facing areas in UEC services [22].

### Study period

To ensure records were available for each complete calendar year, including data on SHSC contacts for the year prior to the beginning of the study period, we examined UEC service usage for 2013, 2014, 2015 and 2016 for Ambulance callouts, Type 1 A&E attendances, APC spells, and 2014, 2015 and 2016 for NHS 111 calls.

### Study populations

We restricted our study to those patients in the CUREd database with a postcode of residence within the boundaries of the Sheffield Clinical Commissioning Group (SCCG).

We identified two groups of interest for each year of the study within this cohort of UEC services users: (i) users of mental health services (UMHS group), defined as anyone in receipt of care from SHSC during the corresponding calendar year, and (ii) those who were not identified as receiving care from SHSC in the study year or preceding year (UEC-only group). For each study year, those patients who had been in receipt of care from SHSC during the preceding year but not the study year were excluded from the analysis. This implemented a ‘washout’ year for patients moving from the UMHS group to the UEC-only group, which was designed to ensure that those patients were truly no longer in need of support from MH services, rather than simply having a low activity period. Any patients who had a start date but a missing end date for a period of SHSC care were included in the UMHS group for the start year only. We excluded individuals who only used services for learning disabilities or for substance misuse disorders (i.e. drug and/or alcohol misuse). We did not exclude older adult services, on the grounds that these services provide care for people with functional disorders (including anxiety, depression and psychotic disorders) as well as dementia.

We excluded children under 15 years old from this analysis. Patients missing age and sex information were excluded. Finally, inpatient spells were excluded where there were inconsistent admission and discharge dates, or where there was evidence of a missing episode from that spell.

### Denominator populations

The dataset provided details of the full UMHS cohort within the specified geographical boundaries (including UEC service users and non-users). However, data on the non-UMHS cohort contained only details of UEC service users, but not non-users. To compare UEC service usage rates, we estimated the size and demographic structure of the (general) population from which the observed sample of UEC-only patients was drawn. To do this, we used age- and sex- stratified ONS population estimates for each study year [24].

### Primary study outcomes

#### Urgent and Emergency Care (UEC) service use

UEC service use was defined as NHS 111 calls, attendances at a Type 1 A&E department (consultant-led 24-hour services with full resuscitation facilities and designated accommodation for the reception of accident and emergency patients), ambulance callouts and APC spells (i.e. continuous periods of care under a single acute hospital provider following an emergency admission) in the corresponding year of the study period.

Primary outcomes comprised (i) annual age- and sex-standardised number of contacts with each of the four services for each cohort in each study year; (ii) annual age- and sex-standardised number of individuals making at least one contact with each of the four services for each cohort in each study year, and (iii) annual age- and sex-standardised rate ratios of the UMHS and general population rates.

### Secondary outcomes

We also sought to compare the following between the two groups of UEC service users, namely the UMHS cohort and the UEC-only group:

### NHS 111 calls

- Whether a call back was required
- Whether a clinical advisor was required to speak with the patient
- Whether the patient was recommended to attend A&E
- Whether an ambulance was sent for the patient
- Whether the patient was recommended to contact primary care services
- Whether the patient was recommended to self-care
- Whether the patient received a different recommendation

### Ambulance callouts

- Whether the source of the callout was an NHS 111 call
- Whether the source of the callout was a 999 call
- Whether the call was categorised as high urgency (‘red’ or ‘purple’ categories) [25]

### A&E attendances

- Whether the patient arrived by ambulance
- Whether the patient was subsequently admitted to a hospital bed or became a lodged patient of the same health care provider
- Whether the patient was discharged to primary care or did not require follow up treatment
- Whether the patient died in the department
- Whether the attendance was determined to be non-urgent [26]

### Inpatient spells

- Whether the patient experienced a stay of 7 nights or more
- Whether the spell consisted of more than one episode (where an episode is defined as a period of care under a given responsible healthcare professional)

Outcomes were documented as standard in the routine data, except for non-urgent A&E attendance which was calculated according to O’Keeffe et al [26], and length of inpatient spell which was calculated as the difference between the discharge and admission dates.

### Analyses

#### Rates of urgent and emergency care use for people with and without UMHS

Annual rates of NHS 111 calls, ambulance callouts, A&E attendances and acute hospital admissions were compared between the two study groups (UMHS versus UEC-only). To do this, we calculated annual age- and sex-standardised rates and 95% confidence intervals (CIs) of UEC service use among the UMHS and UEC-only populations. We carried this out for:

- Usage rates per 1000 population, i.e., the total contacts for each UEC service within each cohort, and;
- Total number of users per 1000 population within each cohort making at least one contact with each UEC service.

Direct standardisation was used to calculate age- and sex- standardised rates, using ONS population estimates for the Sheffield CCG area as the denominator for the general population as well as the standard population. Age strata of 5 year intervals beginning from age 15 up to 85+ were used in accordance with WHO standards [19]. 95% confidence intervals for standardised rates were calculated using the Normal approximation of the Poisson distribution. This method was deemed appropriate due to the large sample size and large number of events for each analysis [27]. Finally, standardised rate ratios (SRRs) of UMHS and non-UMHS rates and their 95% CIs were calculated [28].

#### Features of UEC service usage

We used logistic regression to calculate odds ratios for each of the key features of the usage of each UEC service described above (secondary outcomes). In all cases these were adjusted for age (continuous) and sex. Socioeconomic status was adjusted for using the Index of Multiple Deprivation (IMD) decile (continuous) for the postcode of residence (for NHS 111 calls, A&E attendances and APC episodes) or the postcode of the incident (ambulance callouts). For A&E attendances and inpatient episodes, the Hospital Frailty Risk Score (HFRS; continuous) was also adjusted for as an indication of general health and comorbidity; this was not available for NHS 111 calls or ambulance callouts [29]. Sensitivity analyses were also carried out on Spell data outcomes using Charlson Comorbidity Index (CCI; continuous) in place of HFRS [30]. There was no missing data for included covariates for any year (Table 1; S1A-S1D Table in S1 File). Random effects to account for clustering at the patient level were considered but not included in the final analyses as preliminary work showed them not to materially affect the results. All analyses were performed using R v4.0.4 in RStudio v1.2.5033 in 64bit Windows Server 2019 [31,32].

**Table 1:** User characteristics for all UEC contact events during the study period.

	Excluded	UEC only	UMHS	All
All events	N = 32,072	N = 1,010,538	N = 205,490	N = 1,248,100
**Age**				
Mean (SD)	59.2 (24.9)	50.8 (23.6)	58.3 (24.4)	52.3 (23.9)
Median [Q1, Q3]	60.0 [36.0, 83.0]	49.0 [29.0, 72.0]	58.0 [36.0, 82.0]	50.0 [30.0, 74.0]
**Sex**				
Male	13,497 (42.1%)	469,747 (46.5%)	88,636 (43.1%)	571,880 (45.8%)
Female	18,575 (57.9%)	540,791 (53.5%)	116,854 (56.9%)	676,220 (54.2%)
**IMD decile**				
Median [Q1, Q3]	2.00 [1.0, 6.0]	3.00 [1.0, 6.0]	2.00 [1.0, 6.0]	3.00 [1.0, 6.0]
**Hospital Frailty Risk Score**				
Median [Q1, Q3]	3.20 [0, 14.0]	0 [0, 0.5]	2.30 [0, 11.3]	0 [0, 2.1]
Missing (Not available for NHS111 or Ambulance calls)	14,854 (46.3%)	473,742 (46.9%)	94,977 (46.2%)	583,573 (46.8%)
**NHS 111 calls**	**N = 7,970**	**N = 203,202**	**N = 45,667**	**N = 256,839**
**Age**				
Mean (SD)	54.9 (26.1)	44.5 (22.6)	53.5 (24.2)	46.4 (23.3)
Median [Q1, Q3]	52.0 [29.0, 81.0]	38.0 [25.0, 62.0]	50.0 [31.0, 78.0]	40.0 [26.0, 66.0]
**Sex**				
Male	2,999 (37.6%)	81,110 (39.9%)	18,559 (40.6%)	102,668 (40.0%)
Female	4,971 (62.4%)	122,092 (60.1%)	27,108 (59.4%)	154,171 (60.0%)
**IMD decile**				
Median [Q1, Q3]	2.00 [1.0, 6.0]	3.00 [1.0, 6.0]	2.00 [1.0, 6.0]	3.00 [1.0, 6.0]
**Hospital Frailty Risk Score**	NA	NA	NA	NA
**Ambulance calls**	**N = 6,884**	**N = 270,540**	**N = 49,310**	**N = 326,734**
**Age**				
Mean (SD)	66.8 (22.9)	56.2 (24.1)	62.3 (23.7)	57.3 (24.2)
Median [Q1, Q3]	75.0 [49.0, 86.0]	57.0 [34.0, 78.0]	69.0 [42.0, 84.0]	59.0 [35.0, 80.0]
**Sex**				
Male	2,906 (42.2%)	131,478 (48.6%)	21,620 (43.8%)	156,004 (47.7%)
Female	3,978 (57.8%)	139,062 (51.4%)	27,690 (56.2%)	170,730 (52.3%)
**IMD decile**				
Median [Q1, Q3]	2.00 [1.0, 6.0]	3.00 [1.0, 6.0]	3.00 [1.0, 6.0]	3.00 [1.0, 6.0]
**Hospital Frailty Risk Score**	NA	NA	NA	NA
**A&E attendances**	**N = 11,076**	**N = 370,553**	**N = 71,571**	**N = 453,200**
**Age**				
Mean (SD)	55.2 (24.5)	47.6 (22.4)	54.8 (24.4)	49.0 (23.0)
Median [Q1, Q3]	52.0 [34.0, 80.0]	45.0 [27.0, 66.0]	51.0 [33.0, 79.0]	46.0 [28.0, 69.0]
**Sex**				
Male	4,980 (45.0%)	184,906 (49.9%)	32,355 (45.2%)	222,241 (49.0%)
Female	6,096 (55.0%)	185,647 (50.1%)	39,216 (54.8%)	230,959 (51.0%)
**IMD decile**				
Median [Q1, Q3]	2.00 [1.00, 5.00]	3.00 [1.00, 6.00]	2.00 [1.00, 5.00]	3.00 [1.00, 6.00]
**Hospital Frailty Risk Score**				
Median [Q1, Q3]	1.90 [0, 11.6]	0 [0, 0]	1.50 [0, 9.50]	0 [0, 0.900]
**Inpatient Spells**	**N = 6,142**	**N = 166,243**	**N = 38,942**	**N = 211,327**
**Age**				
Mean (SD)	63.2 (23.5)	56.9 (22.9)	65.1 (23.1)	58.6 (23.2)
Median [Q1, Q3]	69.0 [43.0, 84.0]	60.0 [35.0, 77.0]	74.0 [46.0, 84.0]	62.0 [37.0, 79.0]
**Sex**				
Male	2,612 (42.5%)	72,253 (43.5%)	16,102 (41.3%)	90,967 (43.0%)
Female	3,530 (57.5%)	93,990 (56.5%)	22,840 (58.7%)	120,360 (57.0%)
**IMD decile**				
Median [Q1, Q3]	2.00 [1.0, 6.0]	3.00 [1.0, 7.0]	3.00 [1.0, 6.0]	3.00 [1.0, 7.0]
**Hospital Frailty Risk Score**				
Median [Q1, Q3]	6.20 [0, 17.8]	0 [0, 2.9]	4.40 [0, 14.2]	0 [0, 4.9]

#### Choice of covariates

IMD and frailty were included as covariates in the secondary analyses due to the probability of their association with the outcomes. It was, however, not feasible to adjust for these factors in the primary analyses. In the case of IMD, this would have resulted in an unwieldy number of strata and resulting small sample size in the analyses, and frailty was not possible to adjust for because is not available in ONS population estimates. All included covariates were available as standard in the CUREd database.

## Results

**Fig 1:**
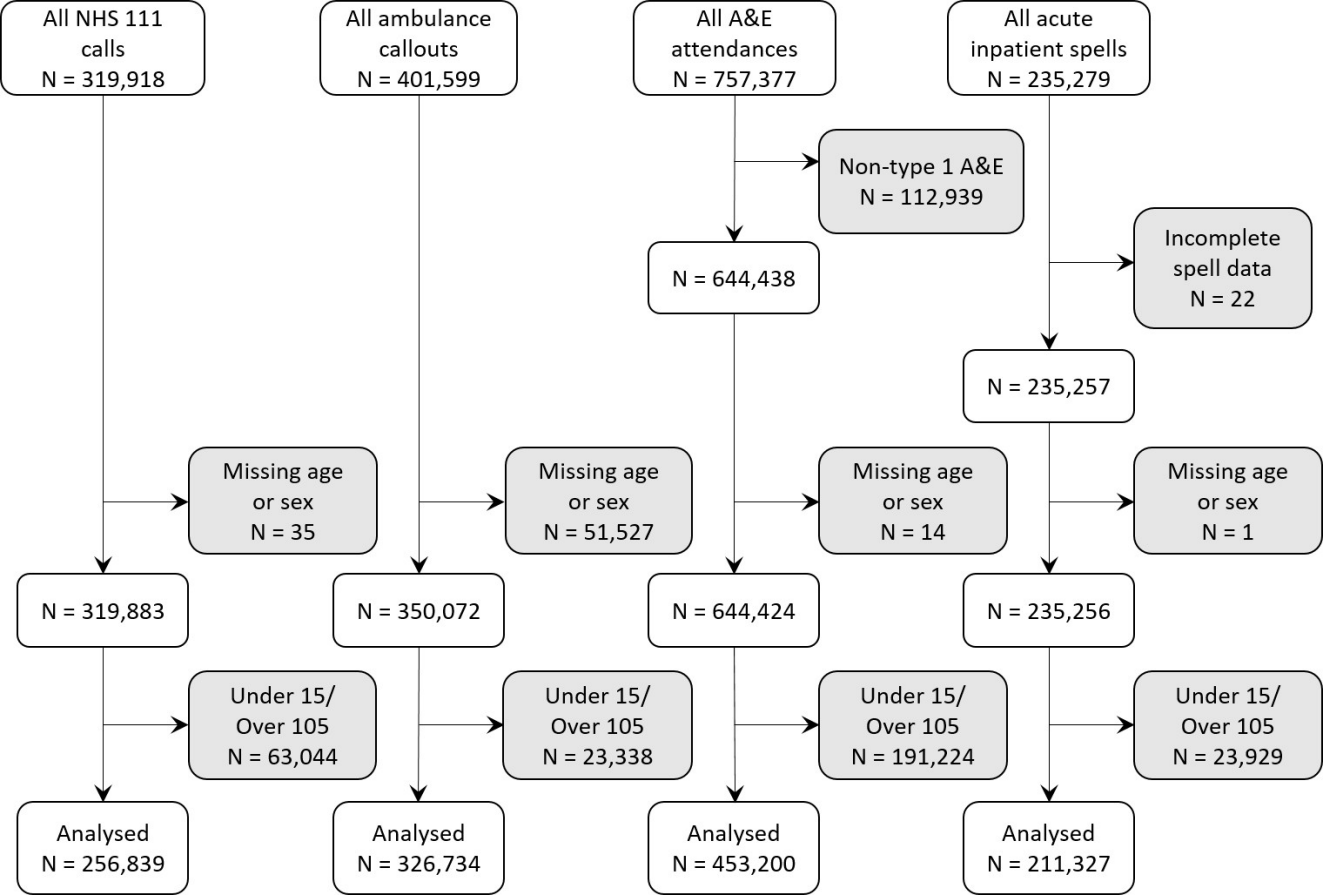
Flow diagram showing exclusions of UEC service records for each service.

After exclusions, during the whole study period there were 256,839 NHS 111 calls by 123,403 patients; 326,734 ambulance callouts by 222,709 patients; 453,200 A&E attendances by 206,987 patients; and 211,327 provider spells by 103,099 patients (Fig 1).

The UMHS cohort comprised 16,642 patients (55.4% female) in 2013; 17,190 patients (55.6% female) in 2014; 18,078 patients (55.6% female) in 2015; and 18,312 patients (54.8% female) in 2016. ONS population estimates for those aged 15+ for the Sheffield CCG area were 464,088 (50.8% female) in 2013; 466,883 (50.8% female) in 2014; 471,897 (50.7% female) in 2015; and 475,898 (50.6% female) in 2016.

The characteristics of service users at each UEC contact for each cohort of service users are shown in Table 1. This also includes details of contacts made by individuals who were excluded from further analysis due to having been a member of the UMHS in the previous year but not in the study year of interest. Generally, those in the UMHS cohort were older than those in the UEC-only cohort. These differences were most marked for ambulance call outs and A&E attendances but were consistent across all UEC services. The two cohorts were broadly similar in deprivation scores, though the UMHS group of A&E attenders appeared to be more deprived than the UEC-only group in this setting. Frailty scores were available for those who attended A&E and for those admitted to acute inpatient beds, where scores for those in the UMHS cohort were higher than for those in the UEC-only group. Frailty scores were highest among those admitted.

**Fig 2:**
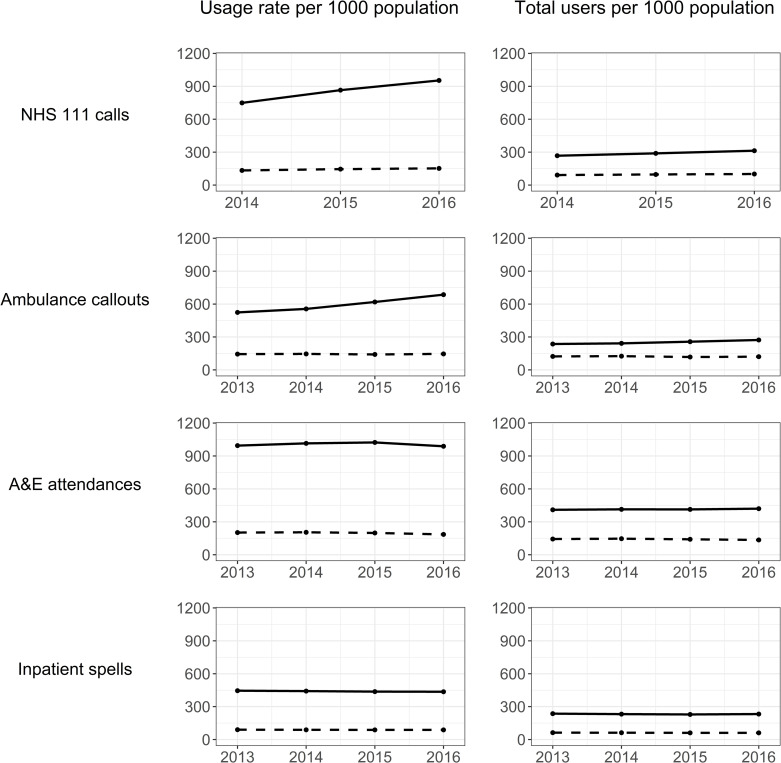
Age & sex standardised usage rates and total users for the UMHS cohort (solid lines) and UEC-only group (dashed lines), per 1000 population for each UEC service by study year.

Crude usage rates are presented S1E Table in S1 File. Age and sex standardised rates indicated that in all study years, the UMHS cohort made greater use of all four UEC services than the UEC-only group (Tables 2 and 3, and Fig 2). Standardised rate ratios were larger for usage rates than for total numbers of users (per 1000 population), and for ambulance callouts and NHS 111 calls than for A&E attendances or hospital admissions. Usage rates per 1000 population were around 6 times higher in the UMHS cohort for NHS 111 calls, 3–5 times higher for ambulance callouts, and 5 times higher for A&E attendance and hospital admissions, respectively, compared with the UEC-only cohort. When overall numbers of users were studied, between-group differences (with higher rates in the UMHS cohort) were around 2 to 4-fold for all UECs. The UMHS cohort therefore consistently made greater use of all UEC services across all study years, and these differences became more pronounced over time for 111 calls, ambulance callouts and A&E attendances.

**Table 2:** Age & sex standardised usage rates (95% confidence intervals) per 1000 population for each UEC service by study year, and comparison as standardised rate ratio (SRR).

Usage rate per 1000 population					Standardised rate ratio	
UMHS			UEC-only			
**NHS 111 calls**						
**2014**	749.53	(734.72, 764.35)	133.23	(132.18, 134.28)	5.63	(5.51, 5.75)
**2015**	865.57	(850.23, 880.90)	145.26	(144.17, 146.34)	5.96	(5.85, 6.08)
**2016**	953.71	(938.05, 969.37)	152.25	(151.14, 153.36)	6.26	(6.15, 6.38)
**Ambulance callouts**						
**2013**	524.06	(511.83, 536.30)	144.13	(143.04, 145.22)	3.64	(3.55, 3.73)
**2014**	556.03	(543.72, 568.33)	145.49	(144.40, 146.59)	3.82	(3.73, 3.91)
**2015**	618.97	(606.40, 631.54)	140.76	(139.69, 141.83)	4.40	(4.30, 4.49)
**2016**	686.01	(673.01, 699.01)	145.62	(144.53, 146.70)	4.71	(4.62, 4.81)
**A&E attendances**						
**2013**	994.71	(977.33, 1012.09)	201.73	(200.44, 203.02)	4.93	(4.84, 5.02)
**2014**	1014.85	(997.73, 1031.98)	204.14	(202.84, 205.43)	4.97	(4.88, 5.06)
**2015**	1023.15	(1006.57, 1039.74)	198.05	(196.78, 199.32)	5.17	(5.08, 5.26)
**2016**	988.64	(972.76, 1004.53)	185.26	(184.04, 186.48)	5.34	(5.25, 5.43)
**Inpatient spells**						
**2013**	445.12	(433.99, 456.26)	89.56	(88.70, 90.42)	4.97	(4.84, 5.11)
**2014**	441.30	(430.57, 452.03)	88.69	(87.84, 89.55)	4.98	(4.85, 5.11)
**2015**	436.99	(426.65, 447.33)	87.79	(86.94, 88.64)	4.98	(4.85, 5.11)
**2016**	435.33	(425.22, 445.44)	87.92	(87.08, 88.76)	4.95	(4.83, 5.08)

**Table 3:** Age & sex standardised usage proportion of users (95% confidence intervals) per 1000 population for each UEC service by study year, and comparison as standardised rate ratio (SRR).

Total users per 1000 population					Standardised rate ratio	
UMHS			UEC-only			
**NHS 111 calls**						
**2014**	267.80	(259.08, 276.52)	91.20	(90.34, 92.07)	2.94	(2.84, 3.04)
**2015**	289.32	(280.58, 298.06)	96.96	(96.07, 97.85)	2.98	(2.89, 3.08)
**2016**	313.01	(304.14, 321.89)	100.90	(99.99, 101.8)	3.10	(3.01, 3.20)
**Ambulance callouts**							
**2013**	236.10	(227.9, 244.3)	123.06	(122.05, 124.07)	1.92	(1.85, 1.99)
**2014**	242.17	(234.05, 250.28)	125.30	(124.28, 126.31)	1.93	(1.87, 2.00)
**2015**	257.10	(248.96, 265.23)	117.24	(116.26, 118.21)	2.19	(2.12, 2.27)
**2016**	272.67	(264.52, 280.82)	120.70	(119.71, 121.69)	2.26	(2.19, 2.33)
**A&E attendances**							
**2013**	409.37	(398.26, 420.48)	143.20	(142.11, 144.29)	2.86	(2.78, 2.94)
**2014**	413.61	(402.71, 424.5)	145.79	(144.7, 146.89)	2.84	(2.76, 2.92)
**2015**	413.13	(402.61, 423.65)	140.87	(139.8, 141.94)	2.93	(2.86, 3.01)
**2016**	419.40	(409.08, 429.72)	134.40	(133.36, 135.44)	3.12	(3.04, 3.20)
**Inpatient spells**							
**2013**	235.65	(227.52, 243.79)	62.88	(62.15, 63.6)	3.75	(3.61, 3.89)
**2014**	231.86	(224.06, 239.67)	61.94	(61.22, 62.65)	3.75	(3.61, 3.88)
**2015**	228.43	(220.93, 235.93)	60.72	(60.02, 61.43)	3.76	(3.63, 3.90)
**2016**	232.47	(225.08, 239.87)	60.62	(59.92, 61.32)	3.84	(3.71, 3.97)

The group excluded for a washout year was fairly large in each study year (Table 1), and thus usage rates in this group were also of interest. Standardised rates could not be calculated due to lack of an appropriate denominator. However, unadjusted average contacts per-person were calculated for all cohorts to facilitate a broad comparison and are presented S1f Table in S1 File. These results suggest usage rates of the excluded group on average between those for the UMHS and UEC-only cohorts.

Table 4 shows adjusted odds ratios (ORs) and 95% CIs for key features of UEC usage. Almost all ORs were significant at the 0.001 level. Individuals in the UMHS cohort were more likely to require a call back from NHS 111 and to be referred to a clinically trained advisor when in contact with this service. They were more likely to receive the highest acuity recommendation from NHS 111 (ambulance callout), but also more likely to receive the lowest acuity recommendation (self-care). In keeping with this, while members of the UMHS cohort who received an ambulance callout were more likely to have done so via a call to NHS 111, they were less likely to have directly called 999 or to receive a high-urgency categorisation.

**Table 4:** Adjusted odds ratios (95% confidence intervals) for key features of UEC service use, comparing UMHS cohort and UEC-only group (reference).

**NHS 111 calls**																	
			**2014**							**2015**				**2016**			
Required call back			1.43			(1.36, 1.51)				1.59		(1.52, 1.66)		1.70		(1.64, 1.77)	
Clinical advisor			1.59			(1.52, 1.66)				1.76		(1.69, 1.83)		1.78		(1.71, 1.85)	
Recommend to attend A&E			0.77			(0.70, 0.85)				0.73		(0.67, 0.79)		0.88		(0.82, 0.94)	
Recommend ambulance			1.42			(1.34, 1.49)				1.48		(1.41, 1.56)		1.50		(1.43, 1.57)	
Recommend Primary Care			0.66			(0.64, 0.69)				0.67		(0.64, 0.69)		0.57		(0.55, 0.59)	
Recommend self care			1.38			(1.31, 1.46)				1.45		(1.38, 1.52)		1.62		(1.55, 1.69)	
Other recommendation			1.45			(1.34, 1.57)				1.32		(1.23, 1.42)		1.53		(1.43, 1.64)	
**Ambulance callouts**																	
		**2013**						**2014**			**2015**				**2016**		
111 call		2.04			(1.90, 2.20)			2.14	(2.02, 2.26)		1.91		(1.81, 2.01)		1.96		(1.87, 2.06)
999 call		0.70			(0.67, 0.73)			0.63	(0.61, 0.66)		0.61		(0.59, 0.64)		0.56		(0.54, 0.58)
High urgency		0.76			(0.73, 0.80)			0.85	(0.81, 0.88)		0.83		(0.80, 0.87)		0.80		(0.76, 0.85)
**A&E Attendances**																	
	**2013**						**2014**				**2015**				**2016**		
Arrival by ambulance	2.83			(2.72, 2.94)			2.67		(2.56, 2.77)		2.67		(2.57, 2.77)		2.63		(2.53, 2.74)
Admitted to hospital	1.32			(1.27, 1.38)			1.31		(1.26, 1.36)		1.25		(1.20, 1.30)		1.24		(1.20, 1.29)
Died in department	0.70*			(0.45, 1.07)			0.52†		(0.31, 0.83)		0.59‡		(0.36, 0.94)		0.42		(0.26, 0.65)
Discharged	0.79			(0.76, 0.82)			0.80		(0.77, 0.83)		0.83		(0.80, 0.86)		0.78		(0.75, 0.81)
Low acuity attendance	1.56			(1.48, 1.64)			1.51		(1.43, 1.58)		1.47		(1.40, 1.54)		1.37		(1.31, 1.43)
**Inpatient spells**																	
	**2013**						**2014**				**2015**				**2016**		
Long length of stay (7+ nights)	1.40			(1.33, 1.48)			1.38		(1.31, 1.46)		1.37		(1.30, 1.44)		1.46		(1.38, 1.54)
Multi-episode spell	1.27			(1.20, 1.34)			1.23		(1.17, 1.30)		1.18		(1.12, 1.24)		1.30		(1.23, 1.36)

Almost all were significant at the 0.001 level

* = not significant

† = significant at the 0.01 level

‡ = significant at the 0.05 level.

Among patients attending A&E, those in the UMHS cohort were almost three times as likely to arrive by ambulance as those in the non-UMHS cohort. They were more likely to be admitted to a hospital bed after attending but were also more likely to be judged to have a low acuity problem amenable to treatment in alternative, non-emergency settings. For patients who experienced an acute hospital admission, patients within the UMHS cohort were more likely to experience a long stay (>7 nights) and to experience multiple episodes of care during their stay under different responsible healthcare professionals.

ORs for the same outcomes for the excluded group vs the UEC-only cohort are presented S1G Table in S1 File. These typically show smaller and less significant or non-significant differences than seen in the UMHS vs UEC-only analysis.

## Discussion

### Main findings

We found marked, statistically significant differences in usage rates for all four Urgent and Emergency Care (UEC) services between the Users of Mental Health Services (UMHS) cohort and the UREC-only group. People with a recent or current history of mental health services use were over-represented among UEC users, and they also made more contacts per person on average. These findings, which also showed that UMHS cohort members were more likely to be admitted to hospital, are consistent with previous research [4,14,15], and with studies showing that that frequent A&E attendance is associated with poorer mental health [33,34]. Our results show, for the first time, that higher rates of service use among those with serious mental health difficulties extends across a wider urgent and acute care pathway.

Mental and physical ill health interact in complex ways that involve biological and psychosocial mechanisms [35–37], People with serious mental illnesses such as schizophrenia, bipolar disorder and personality disorders experience a higher burden of physical disease, higher treatment costs and lower life expectancy than those without these conditions [38]. This group consult more often in primary care [15], but receive less physical health care [13] (including fewer planned hospital admissions [14]), and less proactive care (such as annual physical health reviews) [12,13]; improved access to these types of care could help to reduce UEC attendances in this group [11].

Our findings suggest important differences in the way that users of mental health services access and use UEC services. First, our regression analyses suggest that UMHS patients are more likely present to urgent and emergency care services with problems that are more complex than the general population. Those in the UMHS group were more likely to be admitted following an A&E attendance and more likely to have a long and multi-episode hospital stay. They were also more likely to require a call back from NHS 111 and to speak with a clinical advisor and were less likely to be recommended by NHS 111 to visit primary care, where simpler and less urgent problems may be handled. The possibility that a greater proportion of UMHS patients have complex healthcare needs is generally consistent with previous findings [39]. We included Hospital Frailty Risk Score (HFRS) [40] as a covariate in our analyses of inpatient spells and A&E attendances, allowing us to adjust for comorbidity to some extent. Differences in length of stay and number of episodes remained significant between the cohorts after this adjustment. Sensitivity analysis for spell data was performed using Charlson Comorbidity Index in place of HFRS [30], and these differences remained statistically significant (and were in fact more extreme; S1H Table in S1 File). These results suggest that comorbidity alone does not account for all of the excess UEC use in the UMHS group. Likewise, while socio-economic differences may have confounded our findings [41,42], there was no large systematic difference in IMD between our cohorts, and our regression models remained significant whilst including IMD as a covariate.

However, we also found that the UMHS cohort were more likely to present with lower acuity problems than the UEC-only group. They were more likely to be recommended to self-care by NHS 111, less likely to die in A&E, more likely to have an A&E attendance judged as being of low acuity, and less likely to have a high-urgency ambulance callout. Although there is some evidence that low-acuity or non-urgent ambulance usage is higher among patients with a psychiatric diagnosis or poorer mental health [43,44], most studies examining non-urgent use of UEC services have not examined the role of mental ill health [26,45]. Health anxiety may have been more common in the UMHS cohort [46], leading perhaps to more frequent presentation of minor physical health concerns at UEC services. We did not separate UEC usage by reasons for consultation. To do so is challenging, since many contacts may not exclusively be related to physical or mental health concerns, other but a combination of both. Some presentations would have been associated with mental health crises, such as episodes of self-harm, and it is possible that these accounted for some presentations judged as less acute (or serious). However, we cannot exclude diagnostic overshadowing as an explanation for the finding that some people with a history of mental health service use presenting to urgent and acute care were judged to be less in need of urgent treatment [47].

There may be differences between the two populations that we were unable to account or adjust for that may have confounded the association between UMHS and use of UEC services, for example, lifestyle behaviours such as poor diet, smoking and alcohol and substance abuse [48]. These behaviours undoubtedly cause some of the poorer physical health outcomes that may lead to UEC contacts. However, we did not have information about these exposures in our dataset.

It is notable that for ambulance calls and NHS 111 calls, the difference in rates between cohorts increased over the course of the study–this was seen for both individual calls and number of callers. Pressures on health and social care funding over this time period [49] and increasing shortage of GPs [50] may have affected UMHS patients more than the general population [51]. The combination of fewer resources for mental health services (reducing patients’ ability to maintain their physical health) and difficulty accessing primary care may explain increasingly disproportionate UEC usage between cohorts.

### Generalisability

Sheffield has a population of around 550,000 and is the fourth largest city in England (and the 5th largest in the UK). Like all large cities, Sheffield has significant socio-economic and ethnic diversity. Around one-quarter of communities are in the 10% most deprived areas in the country, and around 50% of children are living in poverty in some parts of the city. Of those living in Sheffield, 8% are of Asian 4% of Black ethnicity, respectively. Around 3% of the city’s population are of White Other, Irish, Gypsy or Irish traveller heritage. We believe, therefore, that our findings are generalisable to other urban areas in England, which is where around 84% of the people in England live. However, additional studies are required to explore and confirm this.

### Strengths and limitations

This study is novel in several ways. It is the first of which we are aware to compare rates of UEC usage amongst those with mental ill health and the general population rather than a limited population of hospital users, and to control for cohort demographics by using general population standardisation [4]. This was also the first study of which we are aware to look at both number of contacts and the number of users in each cohort, and to examine a wider UEC pathway. We have examined several years’ worth of data allowing an understanding of trends in usage over time. Additionally, there are no significant providers of serious mental healthcare outside of the NHS in Sheffield, thus the CUREd data is likely to have captured the vast majority of mental healthcare interactions in Sheffield.

However, while we adjusted for age and sex in our primary analysis, we were unable to control for many important factors. In particular, we could not adjust usage rates for either socioeconomic status or comorbidity, which would be likely to impact these estimates. While we accounted for these where possible in our secondary outcomes, this only explored differences between those individuals making use of UEC services in each cohort. We were also unable to consider the reasons for UEC use.

Due to the scarcity of diagnostic and care cluster information in our dataset, we were unable to restrict our sample to those with confirmed diagnoses of the most severe mental disorders. Instead, we defined our mental illness group, the UMHS cohort, according to recent or current use of specialist secondary care mental health services, excluding only those using services for learning disability or substance misuse. Our sample will therefore have included a number of those with less severe or disabling forms of mental illness. However, given historic (and increasing) difficulty in accessing services [52], it is likely that the majority of the UMHS group will have comprised individuals with schizophrenia, bipolar disorder and severe forms of anxiety, depression and personality disorder. We were also unable to separate the UMHS cohort into those with severe mental illness and those with more mild or moderate mental illness. These subgroups are likely to differ in their patterns of UEC usage and in the features of their contacts, and any proposed interventions to reduce UEC use among UMHS patients would need to be tailored to the differing needs of these subgroups, which the current study is unable to elucidate clearly. We also did not have access to GP data, which would have provided additional information on acute care contacts and allowed further contextualisation of our results.

Possible biases in this analysis include those possible in all analyses of observational data, such as misclassification bias, unmeasured confounding and missing data. We are also limited by imprecision in the ONS population estimates used as the standard population for our analysis as well as the UEC-only group denominator [53]. The inclusion of patients open to older adult mental health services will have contributed to the older mean age of the UMHS cohort compared with UEC participants. Although this group were in the minority, their inclusion will have increased the prevalence of long-term physical health problems in the UMHS group. Excluding older adults would, by contrast, have underestimated the need for urgent and emergency care among users of specialist mental health services. Given the size of our dataset, the issues outlined here are unlikely to significantly impact the direction of our findings but may have implications for their magnitude and precision.

It is likely that some presentations, especially in the UHMS group, were due to mental health crises. Only broad information regarding patient symptoms or reason for presentation was available, therefore a detailed examination was not possible. However, the available data indicates that approximately 6% of NHS 111 calls were for “worsening mental health problems” in the UHMS group and around 0.4% in the UEC-only group. “Psychiatric/suicide attempt” was recorded for about 5% of ambulance calls in the UHMS group but also 3–4% of the UEC-only group. “Deliberate self-harm” was recorded for around 1.5% of A&E attendances among the UHMS group and 0.1% of the UEC group. Overall, we estimate that 5–10% of NHS 111 and ambulance contacts, and 2–5% of A&E attendances, were likely to be due to mental health crises in the UHMS group. A small number of presentations in the UEC-only group were also related to mental health problems, reducing the difference between groups in this type of presentation, and any impact on our main findings.

### Conclusions

Our findings suggest that mental health service use is associated with increased UEC service use across a wider UEC pathway. We also found evidence of a bimodal distribution of UMHS patients in terms of their UEC usage, with these patients tending to present with either more complex or lower acuity health concerns than patients in the general population.

The higher UEC use we observed might be addressed by improved community care, more integrated physical and mental health support, and a more proactive approach in primary care. This will, however, need to be tailored carefully. For instance, addressing health anxiety among some mental health service users (who might also be frequent primary care attenders) might be helped to reduce these contacts, whereas in other patients UEC use arises as a result of delaying presentation to primary or community care. If so, distinct preventative interventions will be needed in primary care and community care settings to reduce UEC use and improve patient outcomes. Community care interventions have been shown to improve some health outcomes [54], but more research is required to understand the mechanisms by which such interventions may be beneficial in different contexts.

## Supporting information

S1 File Supplementary tables. (DOCX)

S2 File RECORD checklist. (DOCX)
