# Supplementary material for: Use of urgent, emergency and acute care by mental health service users: A record-level cohort study
Source: PLoS One. 2023 Feb 13;18(2):e0281667. doi: 10.1371/journal.pone.0281667 (PMC9925080; doi:10.1371/journal.pone.0281667)
Supplement: S1 File — (DOCX) [file pone.0281667.s001.docx]

**Supplementary table S1a**: User characteristics for all NHS 111 calls by year

|  | **Excluded** | **General population** | **MH cohort** | **All** |
| --- | --- | --- | --- | --- |
| 2014 | **N=2,177** | **N=62,202** | **N=13,014** | **N=77,393** |
| **Age** |  |  |  |  |
| Mean (SD) | 55.1 (26.0) | 44.6 (22.5) | 55.6 (24.5) | 46.7 (23.4) |
| Median [Q1, Q3] | 52.0 [30.0, 81.0] | 38.0 [25.0, 62.0] | 53.0 [33.0, 80.0] | 41.0 [26.0, 67.0] |
| **Sex** |  |  |  |  |
| Male | 884 (40.6%) | 24,744 (39.8%) | 4,943 (38.0%) | 30,571 (39.5%) |
| Female | 1,293 (59.4%) | 37,458 (60.2%) | 8,071 (62.0%) | 46,822 (60.5%) |
| **IMD decile** |  |  |  |  |
| Median [Q1, Q3] | 2.00 [1.0, 6.0] | 3.00 [1.0, 6.0] | 2.00 [1.0, 6.0] | 3.00 [1.0, 6.0] |
| 2015 | **N=2,611** | **N=68,546** | **N=15,513** | **N=86,670** |
| **Age** |  |  |  |  |
| Mean (SD) | 55.5 (26.0) | 44.5 (22.7) | 53.7 (24.5) | 46.5 (23.5) |
| Median [Q1, Q3] | 54.0 [29.0, 81.0] | 38.0 [25.0, 62.0] | 50.0 [31.0, 79.0] | 40.0 [26.0, 66.0] |
| **Sex** |  |  |  |  |
| Male | 908 (34.8%) | 27,530 (40.2%) | 6,279 (40.5%) | 34,717 (40.1%) |
| Female | 1,703 (65.2%) | 41,016 (59.8%) | 9,234 (59.5%) | 51,953 (59.9%) |
| **IMD decile** |  |  |  |  |
| Median [Q1, Q3] | 2.00 [1.0, 6.0] | 3.00 [1.0, 6.0] | 2.00 [1.0, 5.0] | 3.00 [1.0, 6.0] |
| 2016 | **N=3,182** | **N=72,454** | **N=17,140** | **N=92,776** |
| **Age** |  |  |  |  |
| Mean (SD) | 54.3 (26.2), | 44.3 (22.6), | 51.8 (23.6), | 46.1 (23.2), |
| Median [Q1, Q3] | 50.0 [29.0, 81.0] | 37.0 [25.0, 62.0] | 49.0 [29.0, 75.0] | 40.0 [26.0, 65.0] |
| **Sex** |  |  |  |  |
| Male | 1,207 (37.9%) | 28,836 (39.8%) | 7,337 (42.8%) | 37,380 (40.3%) |
| Female | 1,975 (62.1%) | 43,618 (60.2%) | 9,803 (57.2%) | 55,396 (59.7%) |
| **IMD decile** |  |  |  |  |
| Median [Q1, Q3] | 2.00 [1.0, 5.0] | 3.00 [1.0, 6.0] | 3.00 [1.0, 5.0] | 3.00 [1.0, 6.0] |

**Supplementary table S1b**: User characteristics for all ambulance callouts by year

|  | **Excluded** | **General**  **population** | **MH cohort** | **All** | |
| --- | --- | --- | --- | --- | --- |
| 2013 | **N=1,228** | **N=66,890** | **N=10,487** | **N=78,605** | |
| **Age** |  |  |  |  | |
| Mean (SD) | 64.9 (23.7) | 56.4 (24.3) | 64.0 (23.5) | 57.6 (24.3) | |
| Median [Q1, Q3] | 75.0 [44.0, 84.0] | 58.0 [34.0, 79.0] | 72.0 [44.0, 84.0] | 60.0 [35.0, 80.0] | |
| **Sex** |  |  |  |  | |
| Male | 544 (44.3%) | 32077 (48.0%) | 4563 (43.5%) | 37184 (47.3%) | |
| Female | 684 (55.7%) | 34813 (52.0%) | 5924 (56.5%) | 41421 (52.7%) | |
| **IMD decile (incident)** |  |  |  |  | |
| Median [Q1, Q3] | 2.00 [1.0, 5.0] | 3.00 [1.0, 6.0] | 3.00 [1.0, 6.0] | 3.00 [1.0, 6.0] | |
| 2014 | **N=1,593** | **N=67,928** | **N=11,447** | **N=80,968** | |
| **Age** |  |  |  |  | |
| Mean (SD) | 66.3 (23.3) | 56.0 (24.2) | 63.4 (23.5) | 57.3 (24.3) | |
| Median [Q1, Q3] | 73.0 [48.0, 86.0] | 57.0 [34.0, 78.0] | 71.0 [44.0, 84.0] | 59.0 [35.0, 80.0] | |
| **Sex** |  |  |  |  | |
| Male | 672 (42.2%) | 32,575 (48.0%) | 4,794 (41.9%) | 38,041 (47.0%) | |
| Female | 921 (57.8%) | 35,353 (52.0%) | 6,653 (58.1%) | 42,927 (53.0%) | |
| **IMD decile (incident)** |  |  |  |  | |
| Median [Q1, Q3] | 3.00 [1.0, 6.0] | 3.00 [1.0, 6.0] | 3.00 [1.0, 6.0] | 3.00 [1.0, 6.0] | |
| 2015 | **N=1,843** | **N=66,424** | **N=13,263** | **N=81,530** | |
| **Age** |  |  |  |  | |
| Mean (SD) | 67.9 (22.3) | 56.1 (24.2) | 62.3 (24.1) | 57.3 (24.3) | |
| Median [Q1, Q3] | 76.0 [51.0, 86.0] | 57.0 [34.0, 78.0] | 69.0 [41.0, 84.0] | 59.0 [35.0, 80.0] | |
| **Sex** |  |  |  |  | |
| Male | 749 (40.6%) | 32,486 (48.9%) | 5,685 (42.9%) | 38,920 (47.7%) | |
| Female | 1,094 (59.4%) | 33,938 (51.1%) | 7,578 (57.1%) | 42,610 (52.3%) | |
| **IMD decile (incident)** |  |  |  |  | |
| Median [Q1, Q3] | 2.00 [1.0, 6.0] | 3.00 [1.0, 6.0] | 3.00 [1.0, 6.0] | 3.00 [1.0, 6.0] | |
| 2016 | **N=2,220** | **N=69,298** | **N=14,113** | **N=85,631** |  |
| **Age** |  |  |  |  |  |
| Mean (SD) | 67.4 (22.6) | 56.2 (23.8) | 60.4 (23.4) | 57.2 (23.8) |  |
| Median [Q1, Q3] | 75.0 [50.0, 86.0] | 57.0 [35.0, 78.0] | 62.0 [41.0, 82.0] | 58.0 [36.0, 79.0] |  |
| **Sex** |  |  |  |  |  |
| Male | 941 (42.4%) | 34340 (49.6%) | 6578 (46.6%) | 41859 (48.9%) |  |
| Female | 1279 (57.6%) | 34958 (50.4%) | 7535 (53.4%) | 43772 (51.1%) |  |
| **IMD decile (incident)** |  |  |  |  |  |
| Median [Q1, Q3] | 2.00 [1.0, 6.0] | 3.00 [1.0, 6.0] | 3.00 [1.0, 6.0] | 3.00 [1.0, 6.0] |  |

**Supplementary table S1c**: User characteristics for all A&E attendances by year

|  | **Excluded** | **General**  **population** | **MH cohort** | **All** |
| --- | --- | --- | --- | --- |
| 2013 | **N=2,358** | **N=93,621** | **N=16,602** | **N=112,581** |
| **Age** |  |  |  |  |
| Mean (SD) | 52.1 (24.1) | 47.2 (22.3) | 55.6 (24.3) | 48.6 (22.9) |
| Median [Q1, Q3] | 47.0 [30.0, 77.0] | 45.0 [27.0, 66.0] | 52.0 [34.0, 80.0] | 46.0 [28.0, 68.0] |
| **Hospital frailty score** |  |  |  |  |
| Median [Q1, Q3] | 1.00 [0, 7.5] | 0 [0, 0] | 1.20 [0, 8.5] | 0 [0, 0.5] |
| **Sex** |  |  |  |  |
| Male | 1,133 (48.0%) | 47,306 (50.5%) | 7,598 (45.8%) | 56,037 (49.8%) |
| Female | 1,225 (52.0%) | 46,315 (49.5%) | 9,004 (54.2%) | 56,544 (50.2%) |
| **IMD decile** |  |  |  |  |
| Median [Q1, Q3] | 2.00 [1.0, 5.0] | 3.00 [1.0, 6.0] | 2.00 [1.0, 6.0] | 3.00 [1.0, 6.0] |
| 2014 | **N=2,649** | **N=95,309** | **N=17,749** | **N=115,707** |
| **Age** |  |  |  |  |
| Mean (SD) | 54.6 (24.2) | 47.2 (22.4) | 55.8 (24.2) | 48.7 (22.9) |
| Median [Q1, Q3] | 51.0 [34.0, 79.0] | 45.0 [27.0, 66.0] | 53.0 [34.0, 80.0] | 46.0 [28.0, 68.0] |
| **Hospital frailty score** |  |  |  |  |
| Median [Q1, Q3] | 1.90 [0, 11.5] | 0 [0, 0] | 1.70 [0, 9.6] | 0 [0, 0.8] |
| **Sex** |  |  |  |  |
| Male | 1,253 (47.3%) | 47,607 (50.0%) | 7,843 (44.2%) | 56,703 (49.0%) |
| Female | 1,396 (52.7%) | 47,702 (50.0%) | 9,906 (55.8%) | 59,004 (51.0%) |
| **IMD decile** |  |  |  |  |
| Median [Q1, Q3] | 2.00 [1.0, 5.0] | 3.00 [1.0, 6.0] | 2.00 [1.0, 5.0] | 3.00 [1.0, 6.0] |
| 2015 | **N=2,929** | **N=93,459** | **N=18,859** | **N=115,247** |
| **Age** |  |  |  |  |
| Mean (SD) | 56.3 (24.2) | 48.0 (22.6) | 54.6 (24.7) | 49.3 (23.1) |
| Median [Q1, Q3] | 53.0 [35.0, 80.0] | 46.0 [27.0, 67.0] | 51.0 [32.0, 80.0] | 47.0 [28.0, 69.0] |
| **Hospital frailty score** |  |  |  |  |
| Median [Q1, Q3] | 2.00 [0, 13.0] | 0 [0, 0] | 1.80 [0, 10.2] | 0 [0, 1.1] |
| **Sex** |  |  |  |  |
| Male | 1,245 (42.5%) | 46,345 (49.6%) | 8,469 (44.9%) | 56,059 (48.6%) |
| Female | 1,684 (57.5%) | 47,114 (50.4%) | 10,390 (55.1%) | 59,188 (51.4%) |
| **IMD decile** |  |  |  |  |
| Median [Q1, Q3] | 2.00 [1.0, 5.0] | 3.00 [1.0, 6.0] | 2.00 [1.0, 5.0] | 3.00 [1.0, 6.0] |
| 2016 | **N=3,140** | **N=88,164** | **N=18,361** | **N=109,665** |
| **Age** |  |  |  |  |
| Mean (SD) | 57.0 (25.0) | 48.2 (22.5) | 53.4 (24.2) | 49.3 (23.0) |
| Median [Q1, Q3] | 56.0 [34.0, 82.0] | 46.0 [28.0, 67.0] | 49.0 [32.0, 78.0] | 47.0 [29.0, 69.0] |
| **Hospital frailty score** |  |  |  |  |
| Median [Q1, Q3] | 2.55 [0, 13.3] | 0 [0, 0] | 1.20 [0, 9.7] | 0 [0, 1.1] |
| **Sex** |  |  |  |  |
| Male | 1,349 (43.0%) | 43,648 (49.5%) | 8,445 (46.0%) | 53,442 (48.7%) |
| Female | 1,791 (57.0%) | 44,516 (50.5%) | 9,916 (54.0%) | 56,223 (51.3%) |
| **IMD decile** |  |  |  |  |
| Median [Q1, Q3] | 2.00 [1.0, 5.0] | 3.00 [1.0, 6.0] | 2.00 [1.0, 5.0] | 3.00 [1.0, 6.0] |

**Supplementary table S1d**: User characteristics for all acute inpatient spells by year

|  | **Excluded** | **General**  **population** | | **MH cohort** | | **All** | |  |
| --- | --- | --- | --- | --- | --- | --- | --- | --- |
| 2013 | **N=1,194** | **N=41,564** | | **N=9,258** | | **N=52,016** | |  |
| **Age** |  |  | |  | |  | |  |
| Mean (SD) | 59.8 (23.9) | 56.5 (23.0) | | 64.9 (23.3) | | 58.1 (23.3) | |  |
| Median [Q1, Q3] | 61.0 [39.0, 82.0] | 60.0 [35.0, 76.0] | | 74.0 [45.0, 84.0] | | 62.0 [37.0, 79.0] | |  |
| **Hospital frailty score** |  |  | |  | |  | |  |
| Median [Q1, Q3] | 4.20 [0, 13.4] | 0 [0, 2.20] | | 3.60 [0, 12.6] | | 0 [0, 3.80] | |  |
| **Sex** |  |  | |  | |  | |  |
| Male | 534 (44.7%) | 18,330 (44.1%) | | 3,843 (41.5%) | | 22,707 (43.7%) | |  |
| Female | 660 (55.3%) | 23,234 (55.9%) | | 5,415 (58.5%) | | 29,309 (56.3%) | |  |
| **IMD decile** |  |  | |  | |  | |  |
| Median [Q1, Q3] | 2.00 [1.0, 6.0] | 3.00 [1.0, 7.0] | | 3.00 [1.0, 6.0] | | 3.00 [1.0, 7.0] | |  |
| 2014 | **N=1,471** | **N=41,409** | | **N=9,799** | | **N=52,679** | |  |
| **Age** |  |  | |  | |  | |  |
| Mean (SD) | 62.4 (23.8) | 56.7 (22.9) | | 66.1 (22.7) | | 58.6 (23.2) | |  |
| Median [Q1, Q3] | 67.0 [43.0, 84.0] | 60.0 [35.0, 76.0] | | 75.0 [48.0, 85.0] | | 63.0 [38.0, 79.0] | |  |
| **Hospital frailty score** |  |  | |  | |  | |  |
| Median [Q1, Q3] | 6.20 [0, 17.6] | 0 [0, 2.7] | | 4.60 [0, 14.2] | | 0 [0, 4.7] | |  |
| **Sex** |  |  | |  | |  | |  |
| Male | 638 (43.4%) | 18,185 (43.9%) | | 4,040 (41.2%) | | 22,863 (43.4%) | |  |
| Female | 833 (56.6%) | 23,224 (56.1%) | | 5,759 (58.8%) | | 29,816 (56.6%) | |  |
| **IMD decile** |  |  | |  | |  | |  |
| Median [Q1, Q3] | 2.00 [1.0, 6.0] | 3.00 [1.0, 7.0] | | 3.00 [1.0, 6.0] | | 3.00 [1.0, 7.0] | |  |
| 2015 | **N=1,655** | **N=41,428** | | **N=10,055** | | **N=53,138** | |  |
| **Age** |  |  | |  | |  | |  |
| Mean (SD) | 64.4 (23.0) | 56.9 (22.9) | | 65.1 (23.3) | | 58.6 (23.2) | |  |
| Median [Q1, Q3] | 71.0 [46.0, 84.0] | 60.0 [35.0, 77.0] | | 74.0 [46.0, 84.0] | | 62.0 [37.0, 79.0] | |  |
| **Hospital frailty score** |  |  | |  | |  | |  |
| Median [Q1, Q3] | 6.80 [0.50, 18.9] | 0 [0, 3.2] | | 5.00 [0, 14.8] | | 0 [0, 5.6] | |  |
| **Sex** |  |  | |  | |  | |  |
| Male | 699 (42.2%) | 17,832 (43.0%) | | 4,084 (40.6%) | | 22,615 (42.6%) | |  |
| Female | 956 (57.8%) | 23,596 (57.0%) | | 5,971 (59.4%) | | 30,523 (57.4%) | |  |
| **IMD decile** |  |  | |  | |  | |  |
| Median [Q1, Q3] | 2.00 [1.0, 6.0] | 3.00 [1.0, 7.0] | | 3.00 [1.0, 6.0] | | 3.00 [1.0, 7.0] | |  |
| 2016 | **N=1,822** | | **N=41,842** | | **N=9,830** | | **N=53,494** | |
| **Age** |  | |  | |  | |  | |
| Mean (SD) | 65.0 (23.3) | | 57.3 (22.8) | | 64.1 (23.2) | | 58.9 (23.1) | |
| Median [Q1, Q3] | 73.0 [46.0, 85.0] | | 61.0 [36.0, 77.0] | | 72.0 [45.0, 84.0] | | 63.0 [38.0, 79.0] | |
| **Hospital frailty score** |  | |  | |  | |  | |
| Median [Q1, Q3] | 7.60 [0.50, 20.2] | | 0 [0, 3.20] | | 4.70 [0, 15.1] | | 0 [0, 5.5] | |
| **Sex** |  | |  | |  | |  | |
| Male | 741 (40.7%) | | 17,906 (42.8%) | | 4,135 (42.1%) | | 22,782 (42.6%) | |
| Female | 1,081 (59.3%) | | 23,936 (57.2%) | | 5,695 (57.9%) | | 30,712 (57.4%) | |
| **IMD decile** |  | |  | |  | |  | |
| Median [Q1, Q3] | 2.00 [1.0, 6.0] | | 3.00 [1.0, 7.0] | | 3.00 [1.0, 6.0] | | 3.00 [1.0, 7.0] | |

**Supplementary table S1e**: Crude UEC usage rates and crude proportions of patients accessing UEC services in each year of study.

|  | UMHS  Usage rate (CI) | UMHS  Proportion (CI) | UEC only  Usage rate (CI) | UEC only  Proportion (CI) |
| --- | --- | --- | --- | --- |
| NHS 111 Calls | |  |  |  |
| 2014 | 0.757  (0.744, 0.77) | 0.294  (0.287, 0.301) | 0.110  (0.109, 0.111) | 0.076  (0.075, 0.077) |
| 2015 | 0.858  (0.844, 0.872) | 0.316  (0.309, 0.323) | 0.120  (0.119, 0.121) | 0.080  (0.079, 0.081) |
| 2016 | 0.935  (0.921, 0.949) | 0.331  (0.324, 0.338) | 0.126  (0.125, 0.127) | 0.084  (0.083, 0.085) |
| Ambulance callouts | |  |  |  |
| 2013 | 0.630  (0.618, 0.642) | 0.288  (0.281, 0.295) | 0.119  (0.118, 0.12) | 0.102  (0.101, 0.103) |
| 2014 | 0.666  (0.654, 0.678) | 0.295  (0.288, 0.302) | 0.121  (0.12, 0.122) | 0.104  (0.103, 0.105) |
| 2015 | 0.733  (0.721, 0.745) | 0.302  (0.295, 0.309) | 0.117  (0.116, 0.118) | 0.097  (0.096, 0.098) |
| 2016 | 0.770  (0.757, 0.783) | 0.314  (0.307, 0.321) | 0.121  (0.12, 0.122) | 0.100  (0.099, 0.101) |
| A&E Attendances | |  |  |  |
| 2013 | 0.997  (0.982, 1.012) | 0.431  (0.423, 0.439) | 0.167  (0.166, 0.168) | 0.119  (0.118, 0.12) |
| 2014 | 1.032  (1.017, 1.047) | 0.438  (0.431, 0.445) | 0.169  (0.168, 0.17) | 0.121  (0.12, 0.122) |
| 2015 | 1.043  (1.028, 1.058) | 0.435  (0.428, 0.442) | 0.164  (0.163, 0.165) | 0.117  (0.116, 0.118) |
| 2016 | 1.001  (0.987, 1.015) | 0.437  (0.43, 0.444) | 0.154  (0.153, 0.155) | 0.111  (0.11, 0.112) |
| Inpatient spells | |  |  |  |
| 2013 | 0.556  (0.545, 0.567) | 0.293  (0.286, 0.3) | 0.074  (0.073, 0.075) | 0.052  (0.051, 0.053) |
| 2014 | 0.586  (0.575, 0.597) | 0.303  (0.296, 0.31) | 0.075  (0.074, 0.076) | 0.052  (0.051, 0.053) |
| 2015 | 0.571  (0.56, 0.582) | 0.295  (0.288, 0.302) | 0.074  (0.073, 0.075) | 0.051  (0.05, 0.052) |
| 2016 | 0.549  (0.538, 0.56) | 0.292  (0.285, 0.299) | 0.074  (0.073, 0.075) | 0.051  (0.05, 0.052) |

**Supplementary table S1f**: Average (mean, 95%CI) contacts per patient per year, split by cohort

|  | **2013** | | **2014** | | **2015** | | **2016** |
| --- | --- | --- | --- | --- | --- | --- | --- |
| **NHS 111 calls** | |  | |  | |  | |
| Excluded |  | | 1.91 (1.83, 1.99) | | 2.03 (1.95, 2.11) | | 2.07 (2.00, 2.14) |
| UEC only |  | | 1.46 (1.45, 1.47) | | 1.50 (1.49, 1.51) | | 1.51 (1.50, 1.52) |
| UMHS |  | | 2.57 (2.53, 2.61) | | 2.72 (2.68, 2.76) | | 2.82 (2.78, 2.86) |
| **Ambulance callouts** | |  | |  | |  | |
| Excluded | 1.74 (1.64, 1.84) | | 1.79 (1.70, 1.88) | | 1.99 (1.90, 2.08) | | 2.00 (1.92, 2.08) |
| UEC only | 1.17 (1.16, 1.18) | | 1.16 (1.15, 1.17) | | 1.20 (1.19, 1.21) | | 1.21 (1.20, 1.22) |
| UMHS | 2.19 (2.15, 2.23) | | 2.26 (2.22, 2.30) | | 2.42 (2.38, 2.46) | | 2.45 (2.41, 2.49) |
| **A&E attendances** | |  | |  | |  | |
| Excluded | 1.80 (1.73, 1.87) | | 1.82 (1.75, 1.89) | | 1.87 (1.80, 1.94) | | 1.81 (1.75, 1.87) |
| UEC only | 1.41 (1.40, 1.42) | | 1.40 (1.39, 1.41) | | 1.41 (1.40, 1.42) | | 1.38 (1.37, 1.39) |
| UMHS | 2.31 (2.27, 2.35) | | 2.36 (2.33, 2.39) | | 2.40 (2.37, 2.43) | | 2.29 (2.26, 2.32) |
| **Inpatient spells** | |  | |  | |  | |
| Excluded | 1.60 (1.51, 1.69) | | 1.68 (1.59, 1.77) | | 1.72 (1.64, 1.80) | | 1.74 (1.66, 1.82) |
| UEC only | 1.42 (1.41, 1.43) | | 1.43 (1.42, 1.44) | | 1.45 (1.44, 1.46) | | 1.45 (1.44, 1.46) |
| UMHS | 1.90 (1.86, 1.94) | | 1.92 (1.88, 1.96) | | 1.93 (1.89, 1.97) | | 1.87 (1.83, 1.91) |

**Supplementary table S1g**: Adjusted odds ratios (95% CIs & p values) for key features of UEC service use comparing excluded cohort and UEC only group (reference)

| **NHS 111 calls** | | | | | | | | | | | | | | | | | | | | | | | | | | | | | |
| --- | --- | --- | --- | --- | --- | --- | --- | --- | --- | --- | --- | --- | --- | --- | --- | --- | --- | --- | --- | --- | --- | --- | --- | --- | --- | --- | --- | --- | --- |
|  | | | **2014** | | | | | | | | | | **2015** | | | | | | | | | | **2016** | | | | | | |
| Required call back | | | 1.05 | | | (0.92, 1.19) | | | | | 0.463 | | 1.10 | | | (0.99, 1.22) | | | | 0.087 | | | 1.14 | | | (1.04, 1.25) | | 0.005 | |
| Clinical advisor | | | 1.13 | | | (1.02, 1.25) | | | | | 0.023 | | 1.19 | | | (1.08, 1.30) | | | | <0.001 | | | 1.14 | | | (1.04, 1.24) | | 0.004 | |
| Recommend to attend A&E | | | 0.89 | | | (0.73, 1.08) | | | | | 0.259 | | 0.70 | | | (0.57, 0.84) | | | | <0.001 | | | 0.88 | | | (0.75, 1.02) | | 0.104 | |
| Recommend ambulance | | | 1.03 | | | (0.91, 1.17) | | | | | 0.653 | | 1.08 | | | (0.96, 1.21) | | | | 0.222 | | | 1.06 | | | (0.95, 1.17) | | 0.312 | |
| Recommend Primary Care | | | 0.66 | | | (0.61, 0.72) | | | | | <0.001 | | 0.77 | | | (0.71, 0.83) | | | | <0.001 | | | 0.77 | | | (0.71, 0.82) | | <0.001 | |
| Recommend self care | | | 1.15 | | | (1.02, 1.30) | | | | | 0.023 | | 1.01 | | | (0.89, 1.13) | | | | 0.921 | | | 1.04 | | | (0.94, 1.15) | | 0.456 | |
| Other recommendation | | | 2.35 | | | (2.04, 2.69) | | | | | <0.001 | | 2.16 | | | (1.90, 2.45) | | | | <0.001 | | | 2.03 | | | (1.79, 2.29) | | <0.001 | |
| **Ambulance callouts** | | | | | | | | | | | | | | | | | | | | | | | | | | | | | |
|  | | **2013** | | | | | | | | **2014** | | | | | | | | **2015** | | | | | | | **2016** | | | | |
| 111 call | | 1.61 | | (1.30, 1.98) | | | | <0.001 | | 2.15 | | (1.88, 2.45) | | | <0.001 | | | 1.78 | (1.56, 2.02) | | | <0.001 | | | 1.75 | | (1.56, 1.96) | | <0.001 |
| 999 call | | 0.73 | | (0.65, 0.82) | | | | <0.001 | | 0.63 | | (0.57, 0.69) | | | <0.001 | | | 0.64 | (0.58, 0.70) | | | <0.001 | | | 0.60 | | (0.55, 0.66) | | <0.001 |
| High urgency | | 0.94 | | (0.84, 1.06) | | | | 0.325 | | 0.96 | | (0.87, 1.07) | | | 0.480 | | | 1.02 | (0.93, 1.12) | | | 0.710 | | | 1.02 | | (0.91, 1.14) | | 0.692 |
| **A&E Attendances** | | | | | | | | | | | | | | | | | | | | | | | | | | | | | |
|  | **2013** | | | | | | | | **2014** | | | | | | | | **2015** | | | | | | | **2016** | | | | | |
| Arrival by ambulance | 1.75 | | | (1.59, 1.93) | | | <0.001 | | 1.81 | | | (1.64, 1.99) | | <0.001 | | | 1.83 | | (1.67, 2.00) | | <0.001 | | | 1.66 | | | (1.51, 1.81) | | <0.001 |
| Admitted to hospital | 0.96 | | | (0.87, 1.06) | | | 0.387 | | 0.90 | | | (0.82, 0.99) | | 0.036 | | | 1.03 | | (0.94, 1.13) | | 0.526 | | | 0.90 | | | (0.82, 0.98) | | 0.022 |
| Died in department | 0.79 | | | (0.24, 1.92) | | | 0.645 | | 0.30 | | | (0.05, 0.98) | | 0.099 | | | 0.31 | | (0.05, 1.02) | | 0.110 | | | 0.93 | | | (0.46, 1.71) | | 0.830 |
| Discharged | 0.97 | | | (0.88, 1.06) | | | 0.455 | | 0.99 | | | (0.91, 1.08) | | 0.833 | | | 1.01 | | (0.93, 1.09) | | 0.839 | | | 1.03 | | | (0.95, 1.11) | | 0.526 |
| Low acuity attendance | 1.23 | | | (1.08, 1.39) | | | 0.001 | | 1.20 | | | (1.05, 1.36) | | 0.005 | | | 1.32 | | (1.17, 1.48) | | <0.001 | | | 1.12 | | | (1, 1.24) | | 0.049 |
| **Inpatient spells** | | | | | | | | | | | | | | | | | | | | | | | | | | | | | |
|  | **2013** | | | | | | | | **2014** | | | | | | | | **2015** | | | | | | | | **2016** | | | | |
| Long length of stay (7+ nights) | 1.04 | | | | (0.90, 1.20) | | 0.555 | | 1.04 | | | (0.92, 1.18) | | 0.525 | | | 0.95 | | (0.84, 1.07) | | 0.360 | | | | 0.89 | | (0.79, 1.00) | | 0.049 |
| Multi-episode spell | 1.27 | | | | (1.11, 1.45) | | <0.001 | | 0.90 | | | (0.80, 1.02) | | 0.114 | | | 0.94 | | (0.84, 1.05) | | 0.266 | | | | 0.97 | | (0.87, 1.08) | | 0.593 |

**Supplementary table S1h**: Sensitivity analysis – ORs (CIs) for secondary outcomes for inpatient spells using Charlson Comorbidiy Index as covariate in place of HFRS.

| **Inpatient spells** | | | | | | | | |
| --- | --- | --- | --- | --- | --- | --- | --- | --- |
|  | **2013** | | **2014** | | **2015** | | **2016** | |
| Long length of stay (7+ nights) | 1.46 | (1.38, 1.54) | 1.46 | (1.39, 1.54) | 1.45 | (1.38, 1.53) | 1.53 | (1.45, 1.61) |
| Multi-episode spell | 1.29 | (1.22, 1.36) | 1.31 | (1.24, 1.37) | 1.26 | (1.2, 1.32) | 1.35 | (1.29, 1.42) |
